# Hemipiperazinediium bis­(pyridine-2,6-dicarboxyl­ato-κ^3^ *O*,*N*,*O*′)gallate(III) pyridine-2,6-dicarboxylic acid dihydrate

**DOI:** 10.1107/S1600536808029140

**Published:** 2008-09-20

**Authors:** Masoud Rafizadeh, Andya Nemati, Zohreh Derikvand

**Affiliations:** a Faculty of Chemistry, Tarbiat Moallem University, Tehran, Iran

## Abstract

The asymmetric unit of the title compound, (C_4_H_12_N_2_)_0.5_[Ga(pydc)_2_]·pydcH_2_·2H_2_O, where pydcH_2_ is pyridine-2,6-dicarboxylic acid, C_7_H_5_NO_4_, contains one half of a centrosymmetric piperazinediium dication, one anion, one uncoord­inated pydcH_2_ mol­ecule and two uncoordinated water mol­ecules, one of which is disordered over two sites in a 1:1 ratio. In the anion, the Ga^III^ ion is coordinated by four O atoms [Ga—O = 1.9706 (16)–2.0494 (15) Å] and two N atoms [Ga—N = 1.9660 (18) and 1.9709 (17) Å] from two pydc ligands in a distorted octa­hedral geometry. The crystal structure exhibits inter­molecular O—H⋯O, N—H⋯O and O—H⋯N hydrogen bonds and π–π inter­actions [centroid–centroid distances of 3.5359 (13) and 3.6550 (14) Å].

## Related literature

For self-assembling systems involving pydcH_2_, see: Aghabozorg *et al.* (2006*a* ▶,*b* ▶). For related complexes of the pyridine-2,6-dicarboxyl­ate ligand with transition metals, see: Rafizadeh *et al.* (2005 ▶, 2006 ▶); Rafizadeh & Amani (2006 ▶); Aghabozorg *et al.* (2007 ▶, 2008 ▶). For details of the synthesis, see: Sheshmani *et al.* (2006 ▶).
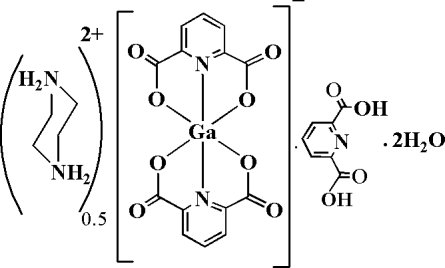


## Experimental

### 

#### Crystal data

(C_4_H_12_N_2_)_0.5_[Ga(C_7_H_3_NO_4_)_2_·C_7_H_5_NO_4_·2H_2_O
*M* *_r_* = 647.16
Triclinic, 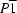

*a* = 8.6434 (4) Å
*b* = 11.8582 (5) Å
*c* = 13.7907 (6) Å
α = 65.7151 (10)°
β = 80.0391 (10)°
γ = 86.9150 (11)°
*V* = 1268.74 (10) Å^3^
*Z* = 2
Mo *K*α radiation
μ = 1.17 mm^−1^
*T* = 120 (2) K
0.25 × 0.20 × 0.18 mm

#### Data collection

Bruker SMART 1000 CCD area-detector diffractometer
Absorption correction: multi-scan (*SADABS*; Sheldrick, 1998 ▶) *T*_min_ = 0.749, *T*_max_ = 0.807
13018 measured reflections
6067 independent reflections
5263 reflections with *I* > 2σ(*I*)
*R*_int_ = 0.024

#### Refinement

*R*[*F* ^2^ > 2σ(*F* ^2^)] = 0.039
*wR*(*F* ^2^) = 0.099
*S* = 1.00
6067 reflections
382 parameters
H-atom parameters constrained
Δρ_max_ = 0.86 e Å^−3^
Δρ_min_ = −0.84 e Å^−3^

### 

Data collection: *SMART* (Bruker, 1998 ▶); cell refinement: *SAINT-Plus* (Bruker, 1998 ▶); data reduction: *SAINT-Plus*; program(s) used to solve structure: *SHELXTL* (Sheldrick, 2008 ▶); program(s) used to refine structure: *SHELXTL*; molecular graphics: *SHELXTL*; software used to prepare material for publication: *SHELXTL*.

## Supplementary Material

Crystal structure: contains datablocks I, global. DOI: 10.1107/S1600536808029140/cv2438sup1.cif

Structure factors: contains datablocks I. DOI: 10.1107/S1600536808029140/cv2438Isup2.hkl

Additional supplementary materials:  crystallographic information; 3D view; checkCIF report

## Figures and Tables

**Table 1:** Hydrogen-bond geometry (Å, °)

*D*—H⋯*A*	*D*—H	H⋯*A*	*D*⋯*A*	*D*—H⋯*A*
N4—H4*B*⋯O13^i^	0.92	1.84	2.754 (3)	169
N4—H4*C*⋯O14	0.92	1.94	2.818 (4)	160
N4—H4*C*⋯O14′	0.92	1.85	2.681 (4)	150
O9—H9*O*⋯O8^ii^	0.89	1.90	2.710 (2)	150
O11—H11*O*⋯O8^ii^	0.87	1.91	2.725 (2)	155
O13—H13*A*⋯O4	0.97	1.88	2.823 (3)	163
O13—H13*B*⋯O2^iii^	0.92	1.84	2.765 (3)	175
O14—H14*A*⋯O10	0.91	1.95	2.798 (5)	153
O14—H14*B*⋯O1^iv^	0.96	2.15	2.974 (5)	143
N4—H4*C*⋯O12^ii^	0.92	2.50	2.863 (3)	104
O9—H9*O*⋯N3	0.89	2.20	2.678 (3)	113
O11—H11*O*⋯N3	0.87	2.22	2.690 (2)	114

Symmetry codes: (i) 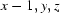; (ii) 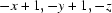; (iii) 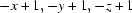; (iv) 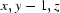.

**Fig. 1.**
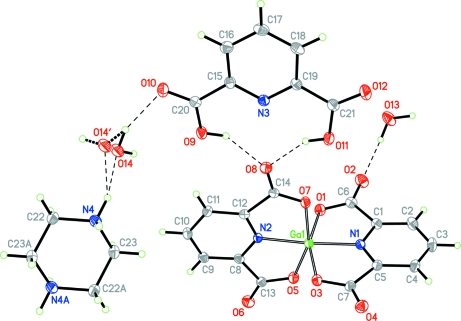
Molecular structure of the title compound, showing the atomic numbering, 50% probability displacement ellipsoids and disordered water molecule. Hydrogen bonds are shown as dashed lines.

## supplementary crystallographic information

### Comment

In continuation of our study of self-assembling systems (pipzH_2_)_3_^2+^[In(pydc)_3_]_2_^3-^.12H_2_O, (pipzH_2_)^2+^[Tl_2_(pydc)_2_Cl_4_(H_2_O)_2_]^2-^.4H_2_O and some others (Aghabozorg *et al.*, 2006*a*,*b*), we present here the crystal structure of the title compound, (I).

In (I) (Fig. 1), all bond lengths and angles are normal and correspond to those observed in the related complexes of pyridine-2,6-dicarboxylate ligand with transition metals (Rafizadeh *et al.*, 2005; Rafizadeh, Mehrabi & Amani, 2006; Rafizadeh & Amani, 2006; Aghabozorg *et al.*, 2007, 2008). In the anion, the angles O1—Ga—O3 [158.69 (6)°], O5—Ga—O7 [158.65 (6)°] and N1—Ga—N2 [171.11 (7)°] indicate that the coordination environment around Ga^III^ ion is a distorted octahedron.

In the crystal, the π–π interactions (Table 1) and extensive three-dimensional network of intermolecular O—H···O, O—H···N and N—H···O hydrogen bonds (Table 2) contribute to the crystal packing stability.

### Experimental

The proton transfer compound (pipzH_2_)(pydcH)_2_.3H_2_O, was prepared by the reaction of pyridine-2,6-dicarboxylic acid, pydcH_2_, with piperazine, pipz, (Sheshmani *et al.*, 2006). The reaction between Ga(NO_3_)_3_.8H_2_O (200.0 mg, 0.5 mmol) in water (25 ml) and the proton transfer compound (pipzH_2 _)(pydcH)_2_.3H_2_O (253.0 mg, 1.0 mmol) in water (25 ml), in a 1:2 molar ratio was carried by slow evaporation of the solvent at room temperature.

### Refinement

The H atoms of the –OH and –NH_2_ groups as well as the water molecule were located in the difference Fourier map and refined in rigid model with fixed thermal (*U*_iso_(H) = 1.2Ueq(O or N) for the –OH and –NH_2_ groups and *U*_iso_(H) = 1.5Ueq(O) for the water molecule) parameters. The H(C) atoms were placed in calculated positions and refined in riding model with fixed thermal parameters (*U*_iso_(H) = 1.2Ueq(C)). The *U*_eq_(O, N or C) are the equivalent thermal parameters of the oxygen, nitrogen and carbon atoms, respectively, to which corresponding H atoms are bonded. One water molecule (O14) was refined as disordered between two positions with the occupancies fixed to 0.5 each.

### Crystal data

**Table:**

(C_4_H_12_N_2_)_0.5_[Ga(C_7_H_3_NO_4_)_2_]·C_7_H_5_NO_4_·2H_2_O	*Z* = 2
*M**_r_* = 647.16	*F*(000) = 660
Triclinic, *P*1	*D*_x_ = 1.694 Mg m^−^^3^
Hall symbol: -P 1	Mo *K*α radiation, λ = 0.71073 Å
*a* = 8.6434 (4) Å	Cell parameters from 6643 reflections
*b* = 11.8582 (5) Å	θ = 2.4–29.9°
*c* = 13.7907 (6) Å	µ = 1.17 mm^−^^1^
α = 65.7151 (10)°	*T* = 120 K
β = 80.0391 (10)°	Prism, colourless
γ = 86.9150 (11)°	0.25 × 0.20 × 0.18 mm
*V* = 1268.74 (10) Å^3^	

### Data collection

**Table:**

Bruker SMART 1000 CCD area-detector diffractometer	6067 independent reflections
Radiation source: fine-focus sealed tube	5263 reflections with *I* > 2σ(*I*)
graphite	*R*_int_ = 0.024
φ and ω scans	θ_max_ = 28.0°, θ_min_ = 1.6°
Absorption correction: multi-scan (SADABS; Sheldrick, 1998a)	*h* = −11→11
*T*_min_ = 0.749, *T*_max_ = 0.807	*k* = −15→15
13018 measured reflections	*l* = −18→18

### Refinement

**Table:**

Refinement on *F*^2^	Primary atom site location: structure-invariant direct methods
Least-squares matrix: full	Secondary atom site location: difference Fourier map
*R*[*F*^2^ > 2σ(*F*^2^)] = 0.039	Hydrogen site location: mixed
*wR*(*F*^2^) = 0.099	H-atom parameters constrained
*S* = 1.00	*w* = 1/[σ^2^(*F*_o_^2^) + (0.043*P*)^2^ + 2.120*P*] where *P* = (*F*_o_^2^ + 2*F*_c_^2^)/3
6067 reflections	(Δ/σ)_max_ = 0.001
382 parameters	Δρ_max_ = 0.86 e Å^−^^3^
0 restraints	Δρ_min_ = −0.84 e Å^−^^3^

### Special details

**Table:**

Geometry. All e.s.d.'s (except the e.s.d. in the dihedral angle between two l.s. planes) are estimated using the full covariance matrix. The cell e.s.d.'s are taken into account individually in the estimation of e.s.d.'s in distances, angles and torsion angles; correlations between e.s.d.'s in cell parameters are only used when they are defined by crystal symmetry. An approximate (isotropic) treatment of cell e.s.d.'s is used for estimating e.s.d.'s involving l.s. planes.
Refinement. Refinement of *F*^2^ against ALL reflections. The weighted *R*-factor *wR* and goodness of fit *S* are based on *F*^2^, conventional *R*-factors *R* are based on *F*, with *F* set to zero for negative *F*^2^. The threshold expression of *F*^2^ > σ(*F*^2^) is used only for calculating *R*-factors(gt) *etc*. and is not relevant to the choice of reflections for refinement. *R*-factors based on *F*^2^ are statistically about twice as large as those based on *F*, and *R*- factors based on ALL data will be even larger.

### Fractional atomic coordinates and isotropic or equivalent isotropic displacement parameters (Å^2^)

**Table:**

	*x*	*y*	*z*	*U*_iso_*/*U*_eq_	Occ. (<1)
Ga1	0.69834 (3)	0.81449 (2)	0.236980 (18)	0.01489 (8)	
O1	0.49972 (19)	0.87108 (15)	0.30323 (13)	0.0197 (3)	
O2	0.3151 (2)	0.80985 (17)	0.45133 (15)	0.0282 (4)	
O3	0.89040 (18)	0.71384 (14)	0.22836 (12)	0.0172 (3)	
O4	1.00566 (19)	0.53668 (15)	0.31985 (13)	0.0221 (3)	
O5	0.82240 (19)	0.95577 (14)	0.22476 (13)	0.0196 (3)	
O6	0.9372 (2)	1.13613 (15)	0.10526 (14)	0.0247 (4)	
O7	0.56973 (18)	0.71043 (14)	0.19088 (12)	0.0175 (3)	
O8	0.49004 (19)	0.70052 (14)	0.04916 (13)	0.0197 (3)	
N1	0.6764 (2)	0.69072 (16)	0.38696 (14)	0.0149 (3)	
N2	0.7106 (2)	0.91756 (16)	0.08145 (14)	0.0136 (3)	
C1	0.5515 (3)	0.6960 (2)	0.45659 (17)	0.0167 (4)	
C2	0.5309 (3)	0.6093 (2)	0.56250 (18)	0.0210 (4)	
H2A	0.4444	0.6131	0.6139	0.025*	
C3	0.6416 (3)	0.5158 (2)	0.59122 (18)	0.0219 (5)	
H3A	0.6310	0.4555	0.6635	0.026*	
C4	0.7666 (3)	0.5101 (2)	0.51542 (17)	0.0192 (4)	
H4A	0.8400	0.4452	0.5342	0.023*	
C5	0.7816 (3)	0.60203 (19)	0.41114 (17)	0.0156 (4)	
C6	0.4444 (3)	0.8002 (2)	0.40167 (18)	0.0191 (4)	
C7	0.9052 (3)	0.6166 (2)	0.31338 (17)	0.0166 (4)	
C8	0.7917 (2)	1.02317 (19)	0.04205 (17)	0.0153 (4)	
C9	0.8099 (3)	1.0982 (2)	−0.06768 (18)	0.0186 (4)	
H9A	0.8670	1.1745	−0.0976	0.022*	
C10	0.7412 (3)	1.0574 (2)	−0.13246 (18)	0.0203 (4)	
H10A	0.7516	1.1069	−0.2077	0.024*	
C11	0.6578 (3)	0.9454 (2)	−0.08861 (17)	0.0174 (4)	
H11A	0.6120	0.9174	−0.1328	0.021*	
C12	0.6438 (2)	0.87575 (19)	0.02216 (17)	0.0147 (4)	
C13	0.8575 (3)	1.0439 (2)	0.12937 (18)	0.0172 (4)	
C14	0.5601 (2)	0.75183 (19)	0.09115 (17)	0.0151 (4)	
O9	0.58636 (19)	0.23498 (15)	0.14907 (13)	0.0210 (3)	
H9O	0.5960	0.2683	0.0773	0.025*	
O10	0.6888 (2)	0.25644 (16)	0.27682 (13)	0.0254 (4)	
O11	0.6148 (2)	0.53088 (15)	−0.19160 (13)	0.0217 (3)	
H11O	0.6047	0.4592	−0.1367	0.026*	
O12	0.7671 (2)	0.69899 (16)	−0.25682 (14)	0.0280 (4)	
N3	0.7038 (2)	0.45536 (16)	0.00267 (14)	0.0157 (3)	
C15	0.7399 (2)	0.4193 (2)	0.10158 (17)	0.0167 (4)	
C16	0.8334 (3)	0.4892 (2)	0.12957 (18)	0.0195 (4)	
H16A	0.8564	0.4600	0.2007	0.023*	
C17	0.8921 (3)	0.6026 (2)	0.05116 (19)	0.0207 (4)	
H17A	0.9560	0.6529	0.0676	0.025*	
C18	0.8560 (3)	0.6413 (2)	−0.05174 (19)	0.0192 (4)	
H18A	0.8950	0.7184	−0.1072	0.023*	
C19	0.7612 (2)	0.56477 (19)	−0.07202 (17)	0.0158 (4)	
C20	0.6712 (3)	0.2969 (2)	0.18329 (18)	0.0182 (4)	
C21	0.7167 (3)	0.6045 (2)	−0.18171 (18)	0.0191 (4)	
N4	0.1327 (2)	0.08293 (17)	0.44706 (15)	0.0178 (4)	
H4B	0.0905	0.1566	0.4452	0.021*	
H4C	0.2365	0.0977	0.4152	0.021*	
C22	0.1227 (3)	−0.0079 (2)	0.56174 (17)	0.0183 (4)	
H22A	0.1770	0.0268	0.6012	0.022*	
H22B	0.1760	−0.0849	0.5644	0.022*	
C23	0.0474 (3)	0.0373 (2)	0.38454 (17)	0.0193 (4)	
H23A	0.0981	−0.0380	0.3808	0.023*	
H23B	0.0525	0.1012	0.3100	0.023*	
O13	0.9874 (2)	0.28616 (16)	0.46782 (16)	0.0303 (4)	
H13A	0.9995	0.3653	0.4063	0.045*	
H13B	0.8844	0.2583	0.4939	0.045*	
O14	0.4588 (4)	0.0692 (4)	0.3846 (3)	0.0273 (6)	0.50
H14B	0.4838	0.0384	0.3297	0.041*	0.50
O14'	0.4459 (4)	0.0981 (4)	0.4270 (3)	0.0273 (6)	0.50
H14C	0.4715	0.0321	0.4851	0.041*	0.50
H14A	0.5272	0.1322	0.3704	0.041*	

### Atomic displacement parameters (Å^2^)

**Table:**

	*U*^11^	*U*^22^	*U*^33^	*U*^12^	*U*^13^	*U*^23^
Ga1	0.01791 (13)	0.01363 (12)	0.01235 (12)	−0.00172 (8)	−0.00268 (8)	−0.00423 (9)
O1	0.0207 (8)	0.0183 (7)	0.0188 (8)	0.0022 (6)	−0.0022 (6)	−0.0070 (6)
O2	0.0233 (9)	0.0275 (9)	0.0299 (9)	0.0030 (7)	0.0045 (7)	−0.0114 (8)
O3	0.0182 (7)	0.0171 (7)	0.0150 (7)	−0.0004 (6)	−0.0020 (6)	−0.0055 (6)
O4	0.0207 (8)	0.0224 (8)	0.0225 (8)	0.0047 (6)	−0.0045 (6)	−0.0085 (7)
O5	0.0254 (8)	0.0175 (7)	0.0172 (7)	−0.0038 (6)	−0.0056 (6)	−0.0070 (6)
O6	0.0277 (9)	0.0187 (8)	0.0281 (9)	−0.0082 (7)	−0.0054 (7)	−0.0086 (7)
O7	0.0219 (8)	0.0161 (7)	0.0135 (7)	−0.0050 (6)	−0.0041 (6)	−0.0039 (6)
O8	0.0232 (8)	0.0178 (7)	0.0201 (8)	−0.0044 (6)	−0.0056 (6)	−0.0082 (6)
N1	0.0157 (8)	0.0153 (8)	0.0133 (8)	−0.0020 (7)	−0.0018 (6)	−0.0055 (7)
N2	0.0136 (8)	0.0127 (8)	0.0129 (8)	0.0003 (6)	−0.0021 (6)	−0.0038 (6)
C1	0.0176 (10)	0.0184 (10)	0.0163 (10)	−0.0015 (8)	0.0001 (8)	−0.0101 (8)
C2	0.0266 (12)	0.0225 (11)	0.0154 (10)	−0.0037 (9)	0.0018 (8)	−0.0107 (9)
C3	0.0341 (13)	0.0176 (10)	0.0129 (10)	−0.0028 (9)	−0.0041 (9)	−0.0045 (8)
C4	0.0255 (11)	0.0163 (10)	0.0162 (10)	−0.0006 (8)	−0.0071 (8)	−0.0054 (8)
C5	0.0182 (10)	0.0137 (9)	0.0159 (10)	−0.0016 (8)	−0.0046 (8)	−0.0062 (8)
C6	0.0204 (11)	0.0180 (10)	0.0211 (10)	−0.0006 (8)	−0.0024 (8)	−0.0105 (8)
C7	0.0178 (10)	0.0176 (10)	0.0162 (10)	−0.0011 (8)	−0.0056 (8)	−0.0076 (8)
C8	0.0147 (9)	0.0128 (9)	0.0178 (10)	−0.0013 (7)	−0.0021 (8)	−0.0057 (8)
C9	0.0180 (10)	0.0146 (10)	0.0201 (10)	−0.0020 (8)	−0.0024 (8)	−0.0038 (8)
C10	0.0207 (11)	0.0210 (11)	0.0144 (10)	−0.0006 (8)	−0.0011 (8)	−0.0030 (8)
C11	0.0183 (10)	0.0191 (10)	0.0150 (10)	0.0008 (8)	−0.0046 (8)	−0.0065 (8)
C12	0.0135 (9)	0.0149 (9)	0.0169 (10)	0.0006 (7)	−0.0030 (7)	−0.0075 (8)
C13	0.0170 (10)	0.0155 (10)	0.0183 (10)	−0.0007 (8)	−0.0035 (8)	−0.0059 (8)
C14	0.0138 (9)	0.0151 (9)	0.0163 (9)	−0.0001 (7)	−0.0024 (7)	−0.0064 (8)
O9	0.0271 (8)	0.0175 (7)	0.0158 (7)	−0.0077 (6)	−0.0034 (6)	−0.0032 (6)
O10	0.0305 (9)	0.0269 (9)	0.0154 (8)	−0.0071 (7)	−0.0040 (7)	−0.0043 (7)
O11	0.0283 (9)	0.0197 (8)	0.0155 (7)	−0.0052 (6)	−0.0070 (6)	−0.0035 (6)
O12	0.0339 (10)	0.0209 (8)	0.0213 (8)	−0.0081 (7)	−0.0045 (7)	0.0002 (7)
N3	0.0173 (9)	0.0148 (8)	0.0161 (8)	−0.0028 (7)	−0.0026 (7)	−0.0071 (7)
C15	0.0166 (10)	0.0190 (10)	0.0145 (9)	0.0000 (8)	−0.0007 (8)	−0.0075 (8)
C16	0.0188 (10)	0.0245 (11)	0.0185 (10)	−0.0008 (8)	−0.0023 (8)	−0.0121 (9)
C17	0.0190 (10)	0.0236 (11)	0.0256 (11)	−0.0048 (8)	−0.0006 (9)	−0.0168 (9)
C18	0.0181 (10)	0.0154 (10)	0.0240 (11)	−0.0032 (8)	0.0007 (8)	−0.0093 (9)
C19	0.0167 (10)	0.0143 (9)	0.0161 (9)	−0.0012 (8)	−0.0006 (8)	−0.0064 (8)
C20	0.0164 (10)	0.0198 (10)	0.0175 (10)	−0.0018 (8)	−0.0020 (8)	−0.0069 (8)
C21	0.0189 (10)	0.0182 (10)	0.0187 (10)	−0.0024 (8)	−0.0015 (8)	−0.0061 (8)
N4	0.0179 (9)	0.0150 (8)	0.0186 (9)	−0.0022 (7)	−0.0011 (7)	−0.0054 (7)
C22	0.0209 (11)	0.0168 (10)	0.0169 (10)	0.0004 (8)	−0.0057 (8)	−0.0057 (8)
C23	0.0239 (11)	0.0184 (10)	0.0148 (10)	−0.0005 (8)	−0.0023 (8)	−0.0061 (8)
O13	0.0278 (9)	0.0159 (8)	0.0394 (10)	−0.0010 (7)	0.0036 (8)	−0.0069 (7)
O14	0.0209 (11)	0.0258 (15)	0.0248 (18)	−0.0049 (10)	−0.0007 (13)	−0.0006 (11)
O14'	0.0209 (11)	0.0258 (15)	0.0248 (18)	−0.0049 (10)	−0.0007 (13)	−0.0006 (11)

### Geometric parameters (Å, °)

**Table:**

Ga1—N1	1.9660 (18)	C12—C14	1.521 (3)
Ga1—O5	1.9706 (16)	O9—C20	1.328 (3)
Ga1—N2	1.9709 (17)	O9—H9O	0.8922
Ga1—O3	2.0073 (16)	O10—C20	1.212 (3)
Ga1—O1	2.0175 (16)	O11—C21	1.334 (3)
Ga1—O7	2.0494 (15)	O11—H11O	0.8710
O1—C6	1.288 (3)	O12—C21	1.208 (3)
O2—C6	1.232 (3)	N3—C19	1.336 (3)
O3—C7	1.282 (3)	N3—C15	1.340 (3)
O4—C7	1.236 (3)	C15—C16	1.390 (3)
O5—C13	1.296 (3)	C15—C20	1.498 (3)
O6—C13	1.218 (3)	C16—C17	1.387 (3)
O7—C14	1.272 (3)	C16—H16A	0.9500
O8—C14	1.234 (3)	C17—C18	1.387 (3)
N1—C5	1.326 (3)	C17—H17A	0.9500
N1—C1	1.332 (3)	C18—C19	1.393 (3)
N2—C8	1.326 (3)	C18—H18A	0.9500
N2—C12	1.333 (3)	C19—C21	1.501 (3)
C1—C2	1.385 (3)	N4—C23	1.492 (3)
C1—C6	1.519 (3)	N4—C22	1.493 (3)
C2—C3	1.399 (3)	N4—H4B	0.9200
C2—H2A	0.9500	N4—H4C	0.9200
C3—C4	1.387 (3)	C22—C23^i^	1.516 (3)
C3—H3A	0.9500	C22—H22A	0.9900
C4—C5	1.391 (3)	C22—H22B	0.9900
C4—H4A	0.9500	C23—C22^i^	1.516 (3)
C5—C7	1.523 (3)	C23—H23A	0.9900
C8—C9	1.390 (3)	C23—H23B	0.9900
C8—C13	1.526 (3)	O13—H13A	0.9680
C9—C10	1.398 (3)	O13—H13B	0.9241
C9—H9A	0.9500	O14—H14B	0.9561
C10—C11	1.393 (3)	O14—H14A	0.9136
C10—H10A	0.9500	O14'—H14C	0.9120
C11—C12	1.393 (3)	O14'—H14A	0.9201
C11—H11A	0.9500		
			
Cg1···Cg1^ii^	3.5359 (13)	Cg2···Cg2^iii^	3.6550 (14)
			
N1—Ga1—O5	108.00 (7)	C10—C11—H11A	121.1
N1—Ga1—N2	171.11 (7)	N2—C12—C11	119.35 (19)
O5—Ga1—N2	80.53 (7)	N2—C12—C14	111.43 (18)
N1—Ga1—O3	79.40 (7)	C11—C12—C14	129.22 (19)
O5—Ga1—O3	92.73 (7)	O6—C13—O5	126.4 (2)
N2—Ga1—O3	98.00 (7)	O6—C13—C8	119.7 (2)
N1—Ga1—O1	79.32 (7)	O5—C13—C8	113.88 (18)
O5—Ga1—O1	92.66 (7)	O8—C14—O7	125.60 (19)
N2—Ga1—O1	103.22 (7)	O8—C14—C12	119.95 (19)
O3—Ga1—O1	158.69 (6)	O7—C14—C12	114.45 (18)
N1—Ga1—O7	93.34 (7)	C20—O9—H9O	111.7
O5—Ga1—O7	158.65 (6)	C21—O11—H11O	111.4
N2—Ga1—O7	78.18 (7)	C19—N3—C15	117.42 (18)
O3—Ga1—O7	91.71 (6)	N3—C15—C16	123.5 (2)
O1—Ga1—O7	90.73 (7)	N3—C15—C20	115.68 (19)
C6—O1—Ga1	115.97 (14)	C16—C15—C20	120.8 (2)
C7—O3—Ga1	116.61 (14)	C17—C16—C15	118.4 (2)
C13—O5—Ga1	116.85 (14)	C17—C16—H16A	120.8
C14—O7—Ga1	116.91 (13)	C15—C16—H16A	120.8
C5—N1—C1	123.75 (19)	C16—C17—C18	118.9 (2)
C5—N1—Ga1	117.85 (14)	C16—C17—H17A	120.6
C1—N1—Ga1	118.25 (15)	C18—C17—H17A	120.6
C8—N2—C12	124.25 (18)	C17—C18—C19	118.6 (2)
C8—N2—Ga1	116.74 (14)	C17—C18—H18A	120.7
C12—N2—Ga1	118.95 (14)	C19—C18—H18A	120.7
N1—C1—C2	119.9 (2)	N3—C19—C18	123.2 (2)
N1—C1—C6	111.16 (18)	N3—C19—C21	116.56 (19)
C2—C1—C6	128.9 (2)	C18—C19—C21	120.18 (19)
C1—C2—C3	117.7 (2)	O10—C20—O9	120.9 (2)
C1—C2—H2A	121.1	O10—C20—C15	122.2 (2)
C3—C2—H2A	121.1	O9—C20—C15	116.82 (19)
C4—C3—C2	120.8 (2)	O12—C21—O11	121.3 (2)
C4—C3—H3A	119.6	O12—C21—C19	122.4 (2)
C2—C3—H3A	119.6	O11—C21—C19	116.24 (19)
C3—C4—C5	118.2 (2)	C23—N4—C22	111.88 (16)
C3—C4—H4A	120.9	C23—N4—H4B	109.2
C5—C4—H4A	120.9	C22—N4—H4B	109.2
N1—C5—C4	119.5 (2)	C23—N4—H4C	109.2
N1—C5—C7	111.65 (18)	C22—N4—H4C	109.2
C4—C5—C7	128.8 (2)	H4B—N4—H4C	107.9
O2—C6—O1	125.6 (2)	N4—C22—C23^i^	110.48 (17)
O2—C6—C1	119.7 (2)	N4—C22—H22A	109.6
O1—C6—C1	114.70 (19)	C23^i^—C22—H22A	109.6
O4—C7—O3	125.8 (2)	N4—C22—H22B	109.6
O4—C7—C5	120.05 (19)	C23^i^—C22—H22B	109.6
O3—C7—C5	114.15 (18)	H22A—C22—H22B	108.1
N2—C8—C9	119.8 (2)	N4—C23—C22^i^	109.99 (18)
N2—C8—C13	111.98 (18)	N4—C23—H23A	109.7
C9—C8—C13	128.25 (19)	C22^i^—C23—H23A	109.7
C8—C9—C10	117.6 (2)	N4—C23—H23B	109.7
C8—C9—H9A	121.2	C22^i^—C23—H23B	109.7
C10—C9—H9A	121.2	H23A—C23—H23B	108.2
C11—C10—C9	121.2 (2)	H13A—O13—H13B	114.1
C11—C10—H10A	119.4	H14B—O14—H14C	137.1
C9—C10—H10A	119.4	H14B—O14—H14A	109.3
C12—C11—C10	117.8 (2)	H14C—O14—H14A	87.3
C12—C11—H11A	121.1	H14C—O14'—H14A	115.4
			
N1—Ga1—O1—C6	−6.27 (15)	N1—C1—C6—O1	−6.8 (3)
O5—Ga1—O1—C6	−114.09 (16)	C2—C1—C6—O1	176.0 (2)
N2—Ga1—O1—C6	165.03 (15)	Ga1—O3—C7—O4	171.78 (17)
O3—Ga1—O1—C6	−9.6 (3)	Ga1—O3—C7—C5	−6.4 (2)
O7—Ga1—O1—C6	87.00 (16)	N1—C5—C7—O4	−174.74 (19)
N1—Ga1—O3—C7	5.46 (15)	C4—C5—C7—O4	3.7 (3)
O5—Ga1—O3—C7	113.27 (15)	N1—C5—C7—O3	3.5 (3)
N2—Ga1—O3—C7	−165.92 (15)	C4—C5—C7—O3	−178.0 (2)
O1—Ga1—O3—C7	8.8 (3)	C12—N2—C8—C9	0.5 (3)
O7—Ga1—O3—C7	−87.62 (15)	Ga1—N2—C8—C9	177.69 (16)
N1—Ga1—O5—C13	175.84 (15)	C12—N2—C8—C13	−178.39 (18)
N2—Ga1—O5—C13	−1.56 (16)	Ga1—N2—C8—C13	−1.2 (2)
O3—Ga1—O5—C13	96.10 (16)	N2—C8—C9—C10	−0.4 (3)
O1—Ga1—O5—C13	−104.53 (16)	C13—C8—C9—C10	178.3 (2)
O7—Ga1—O5—C13	−5.7 (3)	C8—C9—C10—C11	−0.1 (3)
N1—Ga1—O7—C14	−174.98 (16)	C9—C10—C11—C12	0.6 (3)
O5—Ga1—O7—C14	6.5 (3)	C8—N2—C12—C11	0.0 (3)
N2—Ga1—O7—C14	2.31 (15)	Ga1—N2—C12—C11	−177.16 (15)
O3—Ga1—O7—C14	−95.50 (15)	C8—N2—C12—C14	179.72 (18)
O1—Ga1—O7—C14	105.67 (15)	Ga1—N2—C12—C14	2.6 (2)
O5—Ga1—N1—C5	−92.75 (16)	C10—C11—C12—N2	−0.5 (3)
O3—Ga1—N1—C5	−3.32 (15)	C10—C11—C12—C14	179.8 (2)
O1—Ga1—N1—C5	177.91 (16)	Ga1—O5—C13—O6	−177.41 (19)
O7—Ga1—N1—C5	87.80 (16)	Ga1—O5—C13—C8	1.3 (2)
O5—Ga1—N1—C1	91.47 (16)	N2—C8—C13—O6	178.7 (2)
O3—Ga1—N1—C1	−179.09 (17)	C9—C8—C13—O6	0.0 (4)
O1—Ga1—N1—C1	2.14 (15)	N2—C8—C13—O5	−0.1 (3)
O7—Ga1—N1—C1	−87.97 (16)	C9—C8—C13—O5	−178.9 (2)
O5—Ga1—N2—C8	1.50 (15)	Ga1—O7—C14—O8	178.77 (17)
O3—Ga1—N2—C8	−89.95 (16)	Ga1—O7—C14—C12	−1.6 (2)
O1—Ga1—N2—C8	92.02 (16)	N2—C12—C14—O8	179.09 (19)
O7—Ga1—N2—C8	179.97 (16)	C11—C12—C14—O8	−1.2 (3)
O5—Ga1—N2—C12	178.85 (16)	N2—C12—C14—O7	−0.6 (3)
O3—Ga1—N2—C12	87.40 (16)	C11—C12—C14—O7	179.2 (2)
O1—Ga1—N2—C12	−90.63 (16)	C19—N3—C15—C16	−0.2 (3)
O7—Ga1—N2—C12	−2.69 (15)	C19—N3—C15—C20	179.14 (19)
C5—N1—C1—C2	3.5 (3)	N3—C15—C16—C17	0.2 (3)
Ga1—N1—C1—C2	179.03 (16)	C20—C15—C16—C17	−179.1 (2)
C5—N1—C1—C6	−173.91 (19)	C15—C16—C17—C18	−0.3 (3)
Ga1—N1—C1—C6	1.6 (2)	C16—C17—C18—C19	0.3 (3)
N1—C1—C2—C3	−2.1 (3)	C15—N3—C19—C18	0.2 (3)
C6—C1—C2—C3	174.9 (2)	C15—N3—C19—C21	−178.88 (19)
C1—C2—C3—C4	−0.7 (3)	C17—C18—C19—N3	−0.3 (3)
C2—C3—C4—C5	2.1 (3)	C17—C18—C19—C21	178.8 (2)
C1—N1—C5—C4	−2.1 (3)	N3—C15—C20—O10	−176.5 (2)
Ga1—N1—C5—C4	−177.58 (15)	C16—C15—C20—O10	2.9 (3)
C1—N1—C5—C7	176.57 (19)	N3—C15—C20—O9	2.1 (3)
Ga1—N1—C5—C7	1.0 (2)	C16—C15—C20—O9	−178.5 (2)
C3—C4—C5—N1	−0.8 (3)	N3—C19—C21—O12	−176.7 (2)
C3—C4—C5—C7	−179.1 (2)	C18—C19—C21—O12	4.1 (3)
Ga1—O1—C6—O2	−170.02 (19)	N3—C19—C21—O11	4.8 (3)
Ga1—O1—C6—C1	8.8 (2)	C18—C19—C21—O11	−174.3 (2)
N1—C1—C6—O2	172.1 (2)	C23—N4—C22—C23^i^	−57.1 (3)
C2—C1—C6—O2	−5.1 (4)	C22—N4—C23—C22^i^	56.8 (2)

Symmetry codes: (i) −*x*, −*y*, −*z*+1; (ii) −*x*+2, −*y*+1, −*z*; (iii) −*x*+1, −*y*+1, −*z*+1.

### Hydrogen-bond geometry (Å, °)

**Table:**

*D*—H···*A*	*D*—H	H···*A*	*D*···*A*	*D*—H···*A*
N4—H4B···O13^iv^	0.92	1.84	2.754 (3)	169
N4—H4C···O14	0.92	1.94	2.818 (4)	160
N4—H4C···O14'	0.92	1.85	2.681 (4)	150
O9—H9O···O8^v^	0.89	1.90	2.710 (2)	150
O11—H11O···O8^v^	0.87	1.91	2.725 (2)	155
O13—H13A···O4	0.97	1.88	2.823 (3)	163
O13—H13B···O2^iii^	0.92	1.84	2.765 (3)	175
O14—H14A···O10	0.91	1.95	2.798 (5)	153
O14—H14B···O1^vi^	0.96	2.15	2.974 (5)	143
O14'—H14C···O14^vii^	0.91	1.89	2.774 (6)	164
N4—H4C···O12^v^	0.92	2.50	2.863 (3)	104
O9—H9O···N3	0.89	2.20	2.678 (3)	113
O11—H11O···N3	0.87	2.22	2.690 (2)	114
C3—H3A···O11^viii^	0.95	2.48	3.042 (3)	117
C9—H9A···O3^ix^	0.95	2.54	3.341 (3)	143
C17—H17A···O3	0.95	2.57	3.217 (3)	126
C18—H18A···O6^ix^	0.95	2.32	3.026 (3)	130
C22—H22A···O5^iii^	0.99	2.49	3.360 (3)	146
C22—H22B···O2^vi^	0.99	2.50	3.346 (3)	144
C23—H23A···O2^vi^	0.99	2.56	3.391 (3)	142

Symmetry codes: (iv) *x*−1, *y*, *z*; (v) −*x*+1, −*y*+1, −*z*; (iii) −*x*+1, −*y*+1, −*z*+1; (vi) *x*, *y*−1, *z*; (vii) −*x*+1, −*y*, −*z*+1; (viii) *x*, *y*, *z*+1; (ix) −*x*+2, −*y*+2, −*z*.
